# Morphology lies: a case-in-point with a new non-biting midge species from Oriental China (Diptera, Chironomidae)

**DOI:** 10.3897/zookeys.909.39347

**Published:** 2020-02-05

**Authors:** Chao Song, Xinhua Wang, Wenjun Bu, Xin Qi

**Affiliations:** 1 College of Life Sciences, Taizhou University, Taizhou, Zhejiang 318000, China Taizhou University Taizhou China; 2 College of Life Sciences, Nankai University, Tianjin, 300071, China Nankai University Tianjin China

**Keywords:** COI, CAD, *Kiefferulus*, morphology, phylogeny, PGD, taxonomy

## Abstract

Morphological traits are generally indicative of specific taxa, and particularly function as keys in taxonomy and species delimitation. In this study, a non-biting midge species with an *Einfeldia*-like superior volsella makes it hard to accurately determined based on its morphological characteristics. Molecular genes of two ribosomal genes and three protein-encoding genes were compiled to construct a related genera phylogeny and to address the taxonomic issues. Phylogenetic inference clearly supports the undetermined species as belonging to *Kiefferulus*. Therefore, a new species classified in the genus *Kiefferulus* is described and figured as an adult male from Oriental China. The species could be easily distinguished from other species in having an *Einfeldia*-like superior volsella and a triangular tergite IX.

## Introduction

For hundreds of years, taxonomists have been mainly focused on morphological characteristics for classification, taxonomy, and species identification. The most essential part of traditional taxonomy is based on similarities and differences to create systematics. Linnaeus (1753) simplified and standardized the nomenclature into the binomial system of genus and species. However, the system created is mainly based on visible characteristics by taxonomists’ own professional experience, which is unstable and difficult to test. Discoveries and naming of new organisms aim to seek natural groupings with different proxies, such as morphology, genes, ecology, and behavior (Holstein and Luebert 2017). Nevertheless, classification of insects has been based on morphological characteristics to a great extent, which means that one species is deemed to be related with another based on shared characteristics of the same origin (synapomorphies).

With the burgeoning of molecular technology, there have been heated debates among scientists on whether the traditional system should be retained (Garnett and Christidis 2017; Thomson et al. 2018). Some think that the classification of complex organisms is in chaos and hampers species conservation, while others argue that taxonomy is necessary for global species conservation. After more than 250 years of the predominance of comparative morphology in species discovery, advanced methods and technology, especially molecular data, are rapidly expanding the realm of taxonomy (Padial et al. 2010). In addition, molecular information of certain species is increasingly registered or recorded and made available via several global initiatives, such as National Center for Biotechnology Information (NCBI), Barcode of Life Data System (BOLD), and different local barcode libraries (Ratnasingham and Hebert 2007). However, integrative taxonomy requires both detailed morphology description and molecular inference, which is time-consuming. Recently, regarding new species description, it is preferable to provide both morphology and COI barcodes, but COI-based phylogeny inference is unstable and not always convincing. Consequently, with the acceleration of new species descriptions, there would be much likely the peril of erroneous species hypotheses and unstable names (Padial et al. 2010)

Chironomidae is a large family of diverse flies and commonly called non-biting midges. It is the most widely distributed of all aquatic insect families occurring in all zoogeographical region of the world, including Antarctica (Cranston et al. 1989). It also shows adaptions to different extreme niches, surviving at elevations of 5,600 m of Himalaya Mountains (Kohshima 1984) and at more than 1,000 m depth in Lake Baikal (Linnevich 1971).

*Kiefferulus* was described by Goetghebuer (1922) to accommodate *Tanytarsustedipediformis* from Belgium (Chaudhuri and Ghosh 1986). However, it was later recognized as a subgenus of *Pentapedilum* Kiefer by Edwards (1929) and of *Chironomus* Meigen by Townes (1945), after which Hamilton et al. (1969) restored its generic status. The male *Kiefferulus* is easily recognized by its characteristic hypopygium, such as the broadly sickle-shaped superior volsella with numerous long setae on the inner margin and long microtrichia reaching the distal part, and the distal inferior volsella being strongly expanded (Cranston et al. 1989).

Herein, we used sequences from two ribosomal genes (18S and 28S ribosomal DNA), three protein-encoding genes [cytochrome oxidase I (COI), CPSase region of carbamol-phosphate synthase-aspartate transcarbamolylase-dihydroorotase (CAD), and phosphogluconate dehydrogenase (PGD)] to explore the undetermined chironomid species’ systemic position. Through phylogenetic relationships, it is recognized as a new species of *Kiefferulus* based on molecular phylogeny analysis. We also discuss whether morphological traits can be independently used to define species within non-biting midges. Finally, *Kiefferulustrigonum* sp. nov. is presented and described.

## Materials and methods

### Taxon sampling

The morphological nomenclature follows Sæther (1980). The examined specimens were mounted on slides following the procedure by Sæther (1969). Measurements are given as ranges followed by the mean when there are four or more specimens examined. All types are deposited in College of Life Science, Nankai University.

Digital photographs were captured with a Leica DFC420 camera using a Leica DM6000 B compound microscope and under the application of the software Leica Suite at the NTNU university Museum, NTNU (Trondheim, Norway). Photograph post-processing were done in Adobe photoshop and Illustrator (Adobe Inc., California, USA).

### DNA extraction, PCR amplification, sequencing, and alignment

Tissues for total genome DNA extraction were removed from the thorax, heads of adult, and abdomen of larvae. The extraction procedure followed the Qiagen DNeasy Blood and Tissue kit except for elusion buffer ranging from 100–150 µl according to different body sizes. After extraction, the exoskeletons were cleared and mounted to corresponding voucher numbers. We amplified two ribosomal genes (18S and 28S) and four protein coding gene segments including fragments of one mitochondrial gene (COI-3P), two sections of the CPSase region of carbamoylphosphate synthase-aspartate transcarbamoylase-dihydroorotase (CAD1 and CAD4), and phosphogluconate dehydrogenase (PGD). Besides, universal primers LCO1490 and HCO2198 were used for the standard COI barcode sequences.

Polymerase Chain Reaction (PCR) amplifications were done in a 25 µl volume including 12.5 µl 2 × Es Taq MasterMix (CoWin Biotech Co., Beijing, China), 0.625 µl of each primer, 2 µl of template DNA and 9.25 µl deionized H_2_O, or 2.5 µl 10× Takara ExTaq buffer (CL), 2 µl 2.5 mM dNTP mix, 2 µl 25 mM MgCl2, 0.2 µl Takara Ex Taq HS, 1 µl 10 µM of each primer, 2 µl template DNA and 14.3 µl ddH_2_O. PCR was performed on a PowerCylcer Gradient SL (Biometra Gmbh, Göttingen, Germany). For the mitochondrial gene, the program was set as follows: an initial denaturation step of 95 °C for 5 min, then followed by 34 cycles of 94 °C for 30 s, 51 °C for 30 s, 72 °C for 1 min and final extension at 72 °C for 3 min. The program of ribosomal genes and nuclear protein coding genes were referred to Cranston et al. (2012), alternatively for the protein coding genes that a touchdown program: initial denaturation step of 98 °C for 10 s, then 94 °C for 1 min followed by five cycles of 94 °C for 30 s, 52 °C for 30 s, 72 °C for 2 min and 7 cycles of 94 °C for 30 s, 51 °C for 1 min, 72 °C for 2 min and 37 cycles of 94 °C for 30 s, 45 °C for 20 s, 72 °C for 2 min 30 s and one final extension at 72 °C for 3 min. PCR product were confirmed on a 1 % agarose gel and sequenced in both directions with ABI 3730 or ABI 3730XL capillary sequencers at Beijing Genomics Institute Co., Ltd, Beijing, China.

DNA sequences were edited and assembled with BioEdit 7.0.1 (Hall 1999). We applied the appropriate IUPAC code when editing the raw sequences in case of ambiguous bases but use “?” instead of the ambiguity symbol ‘‘N” in the matrix. Sequence matrix of protein coding genes were aligned by their amino acid sequences using Muscle (Edgar 2004) in MEGA7 (Kumar et al. 2016). Introns in CAD and PGD were recognized and deleted according to refence sequences and “GT-AG” rule before analysis. For two ribosomal genes sequences were aligned by muscle and then removed the poorly aligned positions using Glocks online server (http://phylogeny.lirmm.fr/phylo_cgi/one_task.cgi?task_type=gblocks) (Castresana 2000; Dereeper et al. 2008).

### Phylogenetic analysis

Maximum likelihood (ML) trees were constructed in raxml-GUI v1.5b2 (Silvestro and Michalak 2012), with 1000 bootstrap replicates in a rapid bootstrap analysis, using GTR+G+I substitution model with partitions. Bayesian inference analysis (BI) was performed in two parallel runs in MrBayes (Nylander et al. 2004), consisting each of four chains of six million generations with a sampling frequency 1000 generation for one tree and burin of 25%. Partitions were in PartionFinder using greedy search and selected according to aicc (Lanfear et al. 2012). Result was as follows: TRN+I+G for 18S, COI3_ 2; GTR+I+G for COI3p_p1, 28S; GTR+I+G for CAD4_P1, CAD1_P1, PGD_P1; GTR+I+G for CAD1_P2, CAD4_P2, PGD_P2; GTR+I+G for CAD4_P3, PGD_P3; HKY+G for COI3p_P3; HKY+I+G for CAD1_P3. The convergence was checked in Tracer v1.7 (Rambaut et al. 2014) and terminated when ESS were superior to 200 with the initial 25% trees as burn in.

## Results

**Figure 1.**
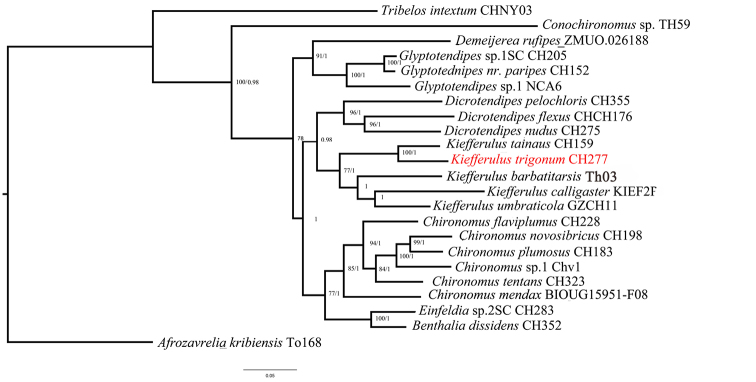
Bayes Inference tree (BI) tree based on the concatenated DNA dataset (18S, 28S, COI3P, CAD1, CAD4, PGD) of *Kiefferulus* and its related genera; *Afrozavrelia* was used as the outgroup. Numbers on branches refer to ML bootstrap values over 75 % and posterior probabilities over 0.95.

The initial sequences of genes are CAD1 909bp, CAD4 846 bp, PGD 747 bp, 18S 933 bp, COI3P 826 bp, and 28S 743 bp (DOI: dx.doi.org/10.5883/DS-KIFFER). To reduce the effects of missing data, we trimmed the beginning and end of the protein coding genes and delete highly variable regions of 18S and 28S and finally concatenated to 4335 bp (CAD1 828 bp, CAD4 760 bp, PGD 747 bp, COI3P 662 bp, 18S 852 bp, 28S 455 bp) (SI). Both ML and BI inference show the same topology (Fig. 1) and agree on the simple phylogenetic scenario: the odd species conflict with the morphotype genus of *Einfeldia* but are clearly supported as species of *Kiefferulus*.

The new species was not identified using morphological taxonomic keys for adult Chironomidae (Cranston et al. 1989). The superior volsella with a large hairy base and a digitiform bare projection was recognized as an important and diagnostic definition of *Einfeldia* sensu lato, which makes it a pre-identification as an *Einfeldia* sp. Nevertheless, the typical superior volsella is not exclusive, and also occurs in *Benthalia*, *Chironomus* (including its subgenera *Chironomus* and *Lobochironomus*), *Conochironomus*, *Glyptotendipes*, and *Tribelos*. From morphological parsimony analysis, these genera sharing similar superior volsella are not closely related (Andersen et al. 2017). Molecular phylogeny of the related genera in this study, and in Cranston et al. (2012) show that *Conochironomus* and *Glyptotendipes* are not closely related to *Einfeldia*. Consequently, generic complexes or species groups with *Einfeldia*-like superior volsella are not genetically monophyletic clades (Fig. 1). Such cases of convergent characters are likely to causes serious problems in phylogenetic analysis, and lead to misplacement of species or genus. The hypothesis of generic diagnosis has raised great confusion within adult taxonomy. The case is not unique in Chironomidae: the marine species *Dicrotendipessinicus* Qi & Lin was suggested as a new genus within the subfamily Chironominae. However, the analysis of genetic data revealed that the marine species nested within the genus *Dicrotendipes* (Qi et al. 2019).

To clearly illustrate the species’ systemic position, it was included in the molecular phylogeny of related genera. Surprisingly, the morphologically identified species fall within the clade of *Kiefferulus* (Fig. 1). Obviously, morphology-based identification conflicts with the molecular phylogeny. Considering the morphological homoplasy and phenotypic changes, we clarified the *Einfeldia*-like species within the genus *Kiefferulus*. While the *Einfeldia*-type superior volsella is unique in *Kiefferulus*, the new species is named as *Kiefferulustrigonum* sp. nov.

When defining a species new to science, almost no taxonomists would test its systemic position, which would be time-consuming and costly. Hierarchical classifications based on appropriate morphological characters provide a main backbone of the life tree, while molecular data provide corroboration, resolution, and support (Scotland et al. 2003). Genera defined and recognized by clear morphological characters as in Chironomidae, such as Wiederholm (1989) for adults, Andersen et al. (2013) for larvae, and Wiederholm (1986) for pupae have not been tested with a full molecular phylogeny. Morphology alone was not enough to make a correct placement especially for some hyper diverse genera or monotypic genera and the traditional taxonomy needs revisions according to molecular phylogeny.

### Taxonomy

#### Kiefferulus trigonum sp. nov.

Taxon classificationAnimaliaDipteraChironomidae

63AA9A50-C1F7-59D1-A6F9-5EDD4406CD69

http://zoobank.org/E880611F-E713-4FCC-9F82-1696F96008A5

**Figure 2.**
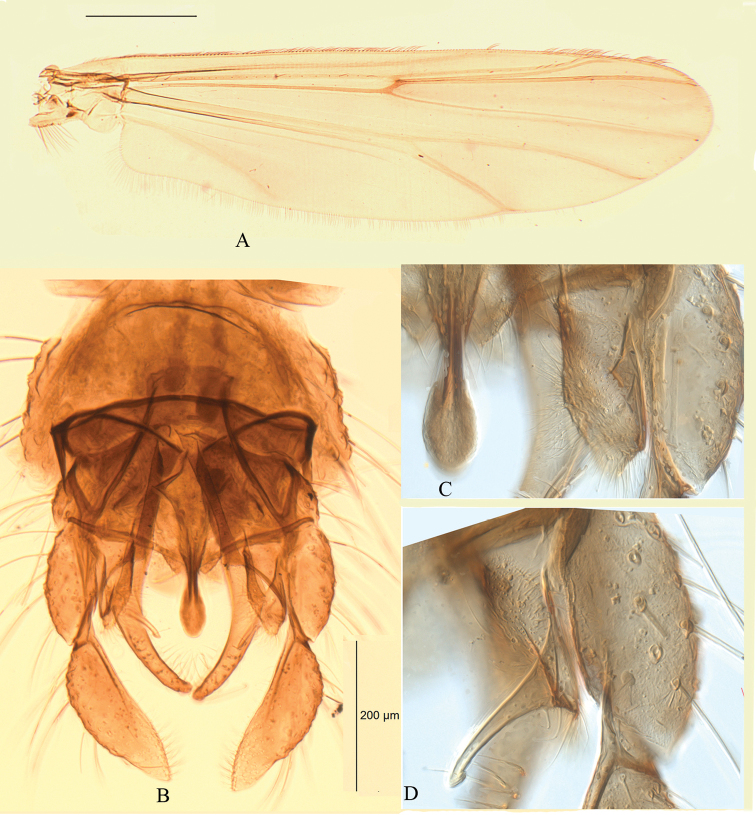
*Kiefferulustrigonum* sp. nov. male **A** wing **B** hypopygium in dorsal view **C** anal point and base of superior volsella **D** projection of superior volsella.

**Figure 3.**
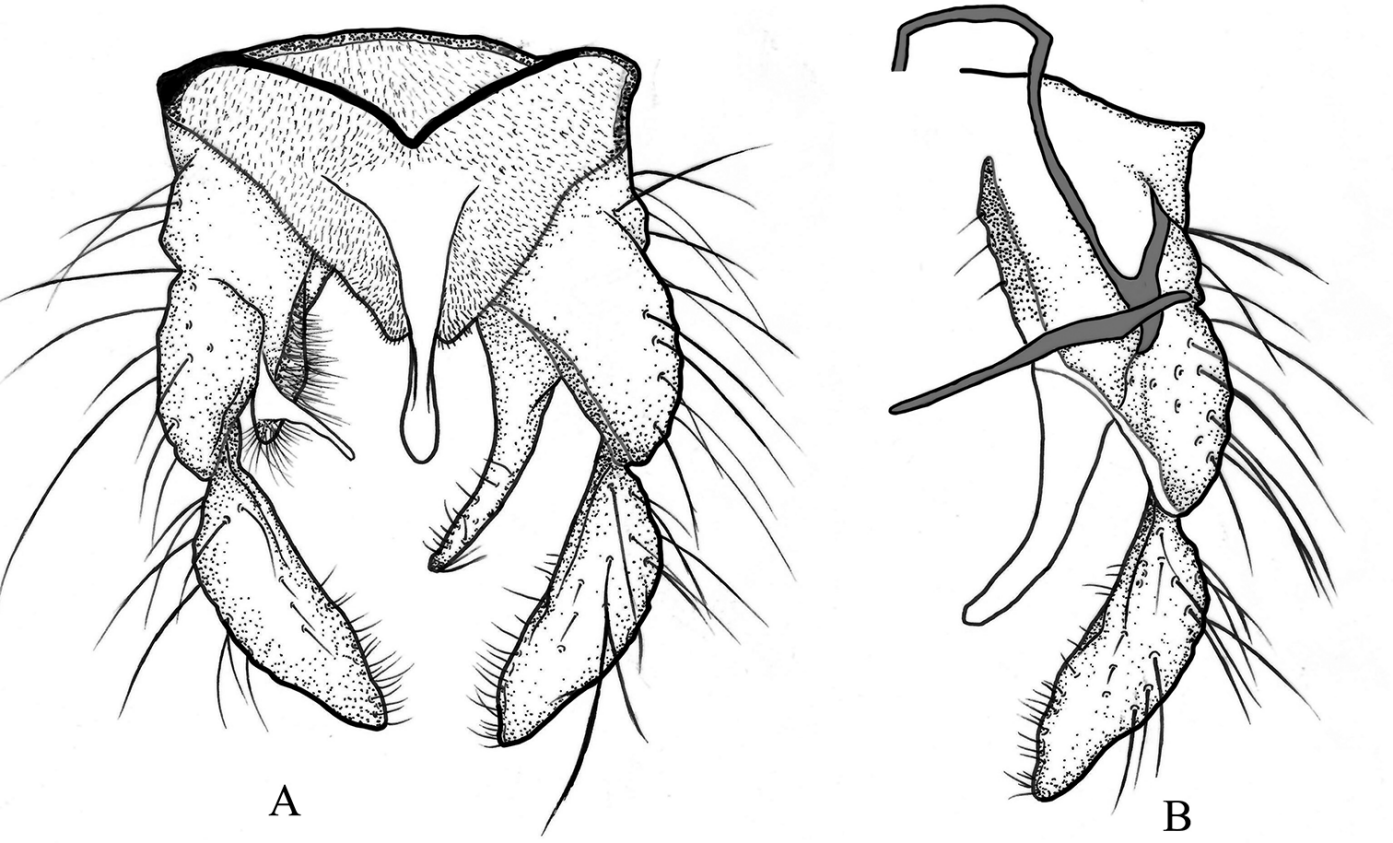
*Kiefferulustrigonum* sp. nov. male **A** hypopygium in dorsal view **B** hypopygium in ventral view.

Figs 2 , 3, BIN: ADG9680

##### Type material.

***Holotype*** (BDN No. 041685) 1♂, China, Fujian Province：Longyan City, Shanghang County, Buyun Town, Qiushan, 25.03N, 116.24E, 6.V.1993, Wang XH, light trap. Paratypes: 1♂ same as holotype; 3♂, Fujian Province: Sanming City, Jianning County, 25. IX.2002, Liu Z, light trap; 1♂, Guangxi Zhuang Autonomous Region: Nanning City, 9. V.1986, Wang XH; 2♂, Guizhou Province: Libo County, 7.VIII.1995, Bu WJ, light trap; 1♂, Hainan Province: Ledong City, Jianfengling National Forest Park, Song C, light trap.

##### Etymology.

From Latin, *trigonum* means triangle, referring to the triangular tergite IX.

##### Diagnostic characters.

The male adults could be obviously distinguished from other *Kiefferulus* species by the triangular IX tergite, superior volsella with projection and basal part of inferior volsella wider than distal part.

##### Description.

Male imago (*N* = 9). Total length 4.78–5.90, 5.30 mm; wing length 2.13–2.85, 2.46 mm; total length/ wing length 1.95–2.38, 2.16; wing length / length of profemur 1.98–2.33, 2.12.

***Coloration.*** Head, thorax and abdomen brown, legs yellowish except distal fore femur and tarsus I light brown.

***Head.*** Frontal tubercle absent. AR 3.07–3.69, 3.25. Temporal setae 15–25, 20; Clypeus with 20–33, 26 setae;

***Palpomere*** lengths (in μm): 38–55; 47; 115–153, 128; 123–163,141; 170–245, 208. Length of 5^th^ palpomere / 3^rd^ palpomere 1.42–2.04, 1.61.

***Wing*** (Fig. 2A) VR 1.07–1.14, 1.10; Brachiolum with 2–3 setae; R with 20–29, 25 setae; R_1_ with 13–21, 17 setae; R_4+5_ with 12–18, 15 setae; squama with 9–19, 15 setae. Anal lobe developed.

***Thorax.*** Dorsocentrals 8–13, 12; acorstichals 20–28, 23; prealars 5–7, 6; scutellum 10–18, 12 setae.

***Legs.*** Tarsomere 1 of Mid and hind leg with 9–20, 14 and 6–17, 10 sensilla chaetica respectively. Front scale bluntly rounded; Spurs of mid tibia 23–38, 29 μm, and 25–33, 29 μm long, of hind tibia 28–38, 32 μm, and 25–35, 29 μm. Width at apex of front tibia 75–98, 85 μm, of mid tibia 83–95, 89 μm, of hind tibia 90–110, 98 μm. Lengths and proportions of legs as the Table 1.

**Table 1.** Length (in μm) and proportions of legs of *Kiefferulustrigonum* sp. nov.

	**fe**	**ti**	**ta_1_**	**ta_2_**	**ta_3_**	**ta_4_**
p_1_	1050–1325, 1161	825–1050, 919	1150–1500, 1322	720–860, 779	630–800, 712	540–690, 611
p_2_	950–1250, 1078	820–1100, 956	520–650, 582	300–380, 337	230–300, 263	160–200, 180
p_3_	1075–1350, 1203	1050–1375, 1194	470–940, 777	420–540, 472	360–480, 418	220–280, 251
	**ta_5_**	**LR**	**BV**	**SV**	**BR**	
p_1_	230–300, 262	1.38–1.52,1.44	1.37–1.49, 1.44	1.53–1.63, 1.57	3.67–6.89, 5.44	
p_2_	110–130, 119	0.58–0.65, 0.61	2.80–3.06, 2.93	3.32–3.67, 3.49	2.32–4.86, 3.53	
p_3_	140–170, 154	0.66–0.69, 0.68	2.35–2.58, 2.47	2.87–3.10, 2. 97	3.24–6.50, 4.09	

***Hypopygium*** (Figs 2B–D, 3). Anal tergite bands medially fused, and median anal tergite seta absent. Anal point basically narrow and apically broaden. Superior volsella with pad-like microchichiose and setose base, with long finger like projection inward to the apex of anal point. Inferior volsella slender, with strong distal setae. Gonocoxite 270–310, 293 μm long, gonostylus 180–210, 198 μm, with numerous distal microtrichia. Phallapodeme 220–250, 232 μm; transverse sternapodeme 100–113, 108 μm. HR 1.42–1.61, 1.49; HV 1.95–2.88, 2.59.

Larva and female unknown.

##### Distribution.

Fujian Province, Guizhou Province, Guangxi Zhuang Autonomous Region, and Hainan Province (Oriental China).

### Remarks

Morphological characters such as the anal point narrow basally, distally broad, the superior volsella with microtrichia, and the gonostylus distally constricted positively and molecular phylogeny provide clues indicating the genus *Kiefferulus*. Morphologically, the new species shows great similarity with *Einfeldia* species with pad-like microtrichose and setose bases and a finger-like projection inwards to the apex of the anal point that clearly distinguishes them from species of *Kiefferulus*.

## Supplementary Material

XML Treatment for Kiefferulus trigonum

## Supplementary materials

Supplementary material 1 The concatenated DNA dataset used for phylogenetic analyses. Data type: NEX file https://binary.pensoft.net/file/377275This dataset is made available under the Open Database License (http://opendatacommons.org/licenses/odbl/1.0/). The Open Database License (ODbL) is a license agreement intended to allow users to freely share, modify, and use this Dataset while maintaining this same freedom for others, provided that the original source and author(s) are credited.Chao Song, Xinhua Wang, Wenjun Bu, Xin Qi
